# Neuronal Function and Dysfunction of *Drosophila* dTDP

**DOI:** 10.1371/journal.pone.0020371

**Published:** 2011-06-01

**Authors:** Meng-Jau Lin, Ching-Wei Cheng, C.-K. James Shen

**Affiliations:** 1 Institute of Molecular Biology, Academia Sinica, Taipei, Taiwan; 2 Institute of Molecular Medicine, National Taiwan University, Taipei, Taiwan; AgroParisTech, France

## Abstract

**Background:**

TDP-43 is an RNA- and DNA-binding protein well conserved in animals including the mammals, *Drosophila*, and *C. elegans*. In mammals, the multi-function TDP-43 encoded by the *TARDBP* gene is a signature protein of the ubiquitin-positive inclusions (UBIs) in the diseased neuronal/glial cells of a range of neurodegenerative diseases including amyotrophic lateral sclerosis (ALS) and frontotemporal lobar degeneration (FTLD-U).

**Methodology/Principal Findings:**

We have studied the function and dysfunction of the *Drosophila* ortholog of the mammalian *TARDBP* gene, *dTDP*, by genetic, behavioral, molecular, and cytological analyses. It was found that depletion of dTDP expression caused locomotion defect accompanied with an increase of the number of boutons at the neuromuscular junctions (NMJ). These phenotypes could be rescued by overexpression of *Drosophila* dTDP in the motor neurons. In contrast, overexpression of dTDP in the motor neurons also resulted in reduced larval and adult locomotor activities, but this was accompanied by a decrease of the number of boutons and axon branches at NMJ. Significantly, constitutive overexpression of dTDP in the mushroom bodies caused smaller axonal lobes as well as severe learning deficiency. On the other hand, constitutive mushroom body-specific knockdown of dTDP expression did not affect the structure of the mushroom bodies, but it impaired the learning ability of the flies, albeit moderately. Overexpression of dTDP also led to the formation of cytosolic dTDP (+) aggregates.

**Conclusion/Significance:**

These data together demonstrate the neuronal functions of dTDP, and by implication the mammalian TDP-43, in learning and locomotion. The effects of mis-expression of dTDP on *Drosophila* NMJ suggest that eukaryotic TDP-43 guards against over development of the synapses. The conservation of the regulatory pathways of functions and dysfunctions of *Drosophila* dTDP and mammalian TDP-43 also shows the feasibility of using the flies as a model system for studying the normal TDP-43 function and TDP-43 proteinopathies in the vertebrates including human.

## Introduction

TDP-43, or the HIV TAR DNA-binding protein 43, is an evolutionarily conserved, 43 kD DNA/RNA-binding protein that functions in transcriptional repression [1], [2], exon 9 skipping of the CFTR pre-mRNA [3], exon 7 inclusion of the SMN pre-mRNA [4], and translational repression [5]. The protein contains two RNA recognition motifs (RRM), RRM1 and RRM2, and a C-terminal domain with glycine-rich (GR) sequence [1]. The RRM domains of TDP-43 preferentially recognize and bind UG-rich RNA and TG-rich DNA [6], [7]. The C-terminus interacts with several members of the heterogeneous ribonucleoprotein (hnRNP) family [8], and it has been suggested to be a prion-like domain in view of its richness in glycine as well as the glutamine and asparagine residues [9]. The majority of the TDP-43 protein is located in the nucleus, and the cytoplasmic TDP-43 molecules reside within the RNA granules and/or P bodies [5].

Interestingly, dysfunction of TDP-43 has been implicated in the pathogenesis of a range of human neurodegenerative diseases, in particular the amyotrophic lateral sclerosis (ALS) and frontotemporal lobar degeneration (FTLD-U). Specifically, the diseased neurons/glial cells of most of the FTLD-U brains and the spinal cord motor neurons of most ALS cases are characterized by the presence of TDP-43-containing, polyubiquitin-positive aggregates or inclusion bodies (UBIs) in the cytoplasm or nuclei. Also, the TDP-43 molecules in the UBIs consist of phosphorylated 45 kD species, high molecular weight polyubiquinated species, and C-terminal fragments of the molecular weights 25 kD and 35 kD, respectively [9], [10], [11], [12], [13], [14], [15]. Although the 25 kD TDP-43 C terminal fragment (CTF), but not the full length TDP-43, forms aggregates much more efficiently in mammalian cell cultures [15], [16], [17], overexpression of the wild type mammalian TDP-43 in transgenic mice or transgenic fruit flies causes neurodegeneration mimicking some of the phenotypes of ALS or FTLD-U [18], [19], [20], [21]. This plus the identifications of more than 30 different TDP-43 mutants associated with ALS [22] suggest that mis-regulation of the metabolism and/or function of TDP-43 is one major cause for the pathogenesis of ALS and FTLD-U.

The pathogenesis of the neurodegenerative diseases with TDP-43 (+) UBIs could be due to toxic gain-of function, loss-of-function of TDP-43, or a combination of both. With respect to this, several studies have implied TDP-43 being a factor important for various neuronal functions. In mouse, mTDP-43 molecules reside in the postsynaptic density (PSD) areas of the dendritic spines. They also form dendritic RNA granules colocalized with the neuronal activity-regulating factors FMRP and Staufen. The above pattern in cultured hippocampal neurons changes upon treatment with various neuronal activity modulating reagents, suggesting the involvement of TDP-43 in the regulation of neuronal plasticity [5]. Consistent with this scenario, CamKII promoter-directed overexpression of mouse mTDP-43 in mice leads to the development of FTLD-U phenotype [20]. Also, Thy1 promoter-directed overexpression of human hTDP-43 in mice causes severe motor neuron dysfunctions, including severe paralysis and spasticity as well as spinal cord neurodegeneration [21]. On the other hand, depletion of *Drosophila* dTDP in the whole bodies of the fruit flies impairs the adult locomotor activities [23]. Depletion of dTDP in the *Drosophila* peripheral sensory neurons also decreases their dendritic branching [24]. Interestingly, overexpression of hTDP-43 in *Drosophila* motor neurons also causes motor dysfunction [18], [19].

The above studies have revealed important insights into the development of FTLD-U-like and ALS-like symptoms by aberrant regulation of TDP-43. In particular, they together have suggested that ALS with TDP-43 (+) UBIs may be in part caused by loss-of-function of TDP-43. In the following, we report our study on the pathogenesis of FTLD-U and ALS using the *Drosophila* as the model system. As seen later, we show that while depletion of the *Drosophila* dTDP and overexpression of dTDP in the *Drosophila* motor neurons both affect the locomotor activity of the flies, the two genetic manipulations exert opposite effects on the development of the boutons at the neuromuscular junctions (NMJ). This suggests a role of dTDP/TDP-43 in the regulation of neuron development. Furthermore, mushroom body-specific depletion or overexpression of dTDP leads to the impairment of the fly learning ability. The latter result together with our recent study of mTDP-43 overexpression in the forebrain of mice [20] suggest that FTLD-U with TDP-43 (+) UBIs is likely caused in part through loss-of-TDP-43 function.

## Results

### Regulation of development, locomotor activity, and NMJ bouton number by *dTDP*

**Figure 1:**
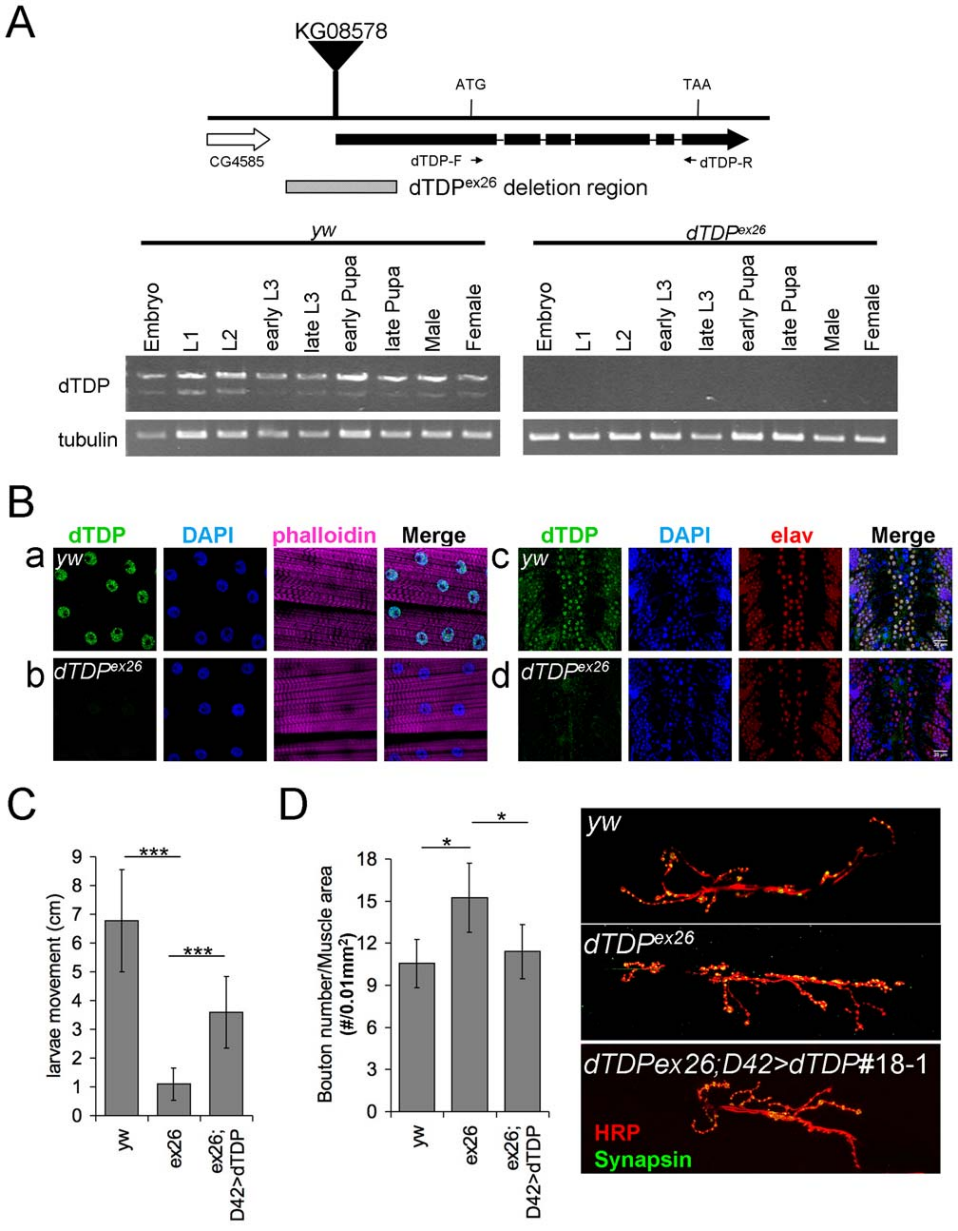
Characteristics of the *dTDP* imprecise excision mutant *dTDP^ex26^*. (A) Organization of the six-exon *dTDP* gene in the *dTDP^ex26^* line. This semi-lethal *dTDP* was generated by imprecise excision of the P-element KG08578 leading to a 932 bp-deletion (the hatched block) in the 5′ end of the *dTDP* gene. The primers used for PCR in the RT-PCR analysis are indicated. Also shown is the most neighboring gene CG4585 located at upstream of dTDP. Lower panels, RT-PCR analysis of *dTDP* gene expression in the wild type (*yw*, left panel) and homozygous *dTDP^ex26^* (right panel). Note the absence of dTDP signal in the *dTDP^ex26^* samples. (B) Immunostaining of the third instar larval segment A3 muscle 6/7 (a,b) and the third instar larval ventral nerve cords (c,d) from *yw* control and *dTDP^ex26^* mutant with anti-dTDP antibody (green). The nuclei were visualized by DAPI (blue), and the actin cytoskeleton was labeled by phalloidin (magenta). The neurons of the ventral nerve cords were also stained with anti-elav (red). Panel rows a and c show the nuclear localization of dTDP in the *yw* control and panel rows b and d show the lack of anti-dTDP signals in the *dTDP^ex26^* mutant. (C) Larval movement assay. The movement of the wandering third instar larvae on the agar plate was monitored for 2 minutes and the average distance of the larval trails was calculated. Note the severe movement defect of the homozygous *dTDP^ex26^* mutant in comparison to the *yw* control, and the partial rescue of this phenotype in *dTDP^ex26^*;* D42>dTDP#18-1* (*N* = 15, ***, *p*<0.0001). (D) Comparison of the bouton number/muscle area of *yw*, *dTDP^ex26^* and *dTDP^ex26^; D42>dTDP#18-1*. The A3 NMJs on muscles 6/7 of the late third instar larvae were co-stained with antibodies against HRP (red) and Synapsin (green), respectively, as exemplified in the right 2 panels. The *dTDP^ex26^* mutant had higher bouton number per unit area of the muscle than the *yw* control, and this phenotype of *dTDP^ex26^* was rescued in *dTDP^ex26^; D42>dTDP#18-1*. *, *p*<0.05. The averages of the total bouton numbers are: *yw*, 67±17 (N = 18); *dTDP^ex26^*, 94±14 (N = 16); *dTDP^ex26^*;* D42>dTDP#18-1*, 75±8 (N = 12). The means of the muscle areas are: *yw*, 63,187 µm^2^; *dTDP^ex26^*, 62,565 µm^2^; *dTDP^ex26^; D42>dTDP#18-1*, 64,310 µm^2^.

The function of *dTDP* in *Drosophila* development was examined by analysis of fly mutants, with null-expression of dTDP. For this, deletion mutations of the *dTDP* gene in the *KG08578* insertion line were generated by imprecise excision. Twelve out of the ninety stocks with excision of the P element from *KG08578* could not survive to the adult stage. One of these recessive lethal *dTDP* lines, *dTDP^ex26^*, was analyzed in details. *dTDP^ex26^* consisted of an imprecise excision of the P-element leading to a 932 bp-deletion in the 5′ end of *dTDP* gene, and no dTDP expression could be detected by RT-PCR analysis at all developmental stages of the homozygous mutant *dTDP^ex26^* flies obtained with use of the green balancer (Fig. 1A). The deletion in *dTDP^ex26^* mutant did not affect the RNA level from the gene CG4585 at upstream of *dTDP* (RT-PCR data not shown). The homozygous *dTDP^ex26^* line was semi-lethal with most of the flies viable from embryonic to early pupal stage but few of them (approximately 10%) eclosed to the adult stage. Most of the flies were trapped in the pupal cases and the survived ones showed weakness in their legs and severe movement defects.

The movement defect was also detected in the larvae of the homozygous *dTDP^ex26^* mutant. As shown in Fig. 1B, immunofluorescence staining of dTDP in the muscle area and the ventral nerve cords of the late third instar larvae of *yw* control showed that dTDP was indeed absent in the homozygous *dTDP^ex26^* mutant. This loss of dTDP expression strongly impaired the locomotive behavior of the larvae by 90%. Quantitatively, the *dTDP^ex26^* mutant displayed significantly shorter moving distance than the control (*p*<0.0001) (Fig. 1 C). To examine whether dTDP played a role in NMJ formation, we dissected late third instar larvae from both the *yw* control and the homozygous *dTDP^ex26^* mutant. The larvae were immunostained with horseradish peroxidase (HRP), which labeled the neuronal membranes, and antibody against synapsin (Syn), a presynaptic protein located in the bouton (Fig. 1D). As seen, interestingly, the number of the synaptic boutons was increased by approximately 35% when normalized to the total muscle areas of muscles 6 and 7 (Fig. 1D). The increase of the NMJ bouton numbers of larvae upon depletion of dTDP was also observed in flies with knockdown of dTDP in the pan-neurons, as described later. Accompanied with the above, the number of the axon branches also increased by 20% as the result of null expression of dTDP (data not shown). Significantly, the phenotypes of the movement dysfunction and increased NMJ bouton numbers could be partially (Fig. 1C) and fully rescued (Fig. 1D), respectively, by specific overexpression of dTDP in the motor neurons. The data of Fig. 1 showed that dTDP was important for *Drosophila* development as well as the locomotor activity of the larvae and adult flies. Furthermore, the latter effect was mediated in part through the regulation of the biogenesis of the *Drosophila* synaptic boutons by dTDP.

### Morphological, cellular, and biochemical characterizations of CNS of *Drosophila* with knockdown or overexpression of dTDP in the mushroom bodies

**Figure 2:**
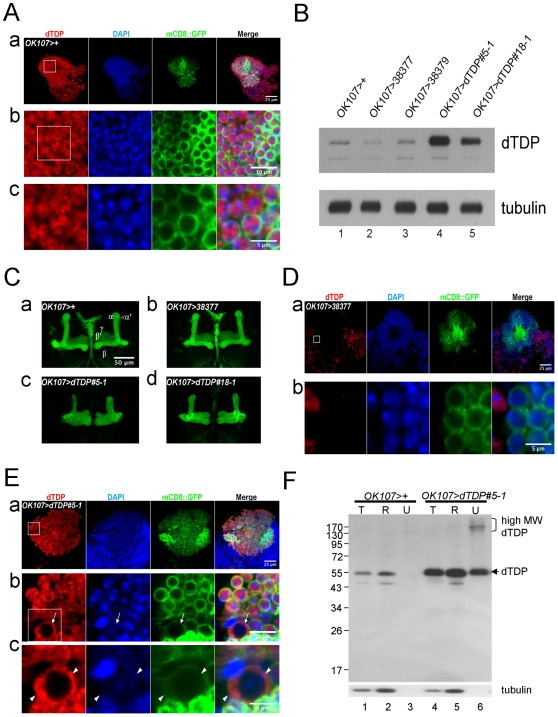
dTDP in the adult brains of 3-day old wild type and mutant *Drosophila*. (A) Distribution of the endogenous dTDP proteins in the mushroom bodies of the *OK107>+* flies as analyzed by immunostaining with anti-dTDP (red), DAPI (blue), and mCD8::GFP (green). (a) a low-magnification photo; (b), higher magnification photo of the boxed area in (a); (c), higher magnification photo of the boxed area in (b). Note the predominant nuclear localization of dTDP. (B) Western blot of the total head protein extracts from the adult flies. The blot was probed with anti-dTDP and anti-tubulin. Note the decreases, by approximately 46% and 23%, of the dTDP levels in the heads of *OK107>38377* (lane 2) and *OK107>38379* (lane 3), respectively, with mushroom body-specific dsRNA-knockdown of dTDP. Also, dTDP levels in the *dTDP*-overexpressing lines *OK107>dTDP#5-1* (lane 4) and *OK107>dTDP#18-1* (lane 5) were 7- and 3 fold, respectively, higher than the *OK107>+* control (lane 1). (C) GFP-lighted morphologies of the mushroom bodies in *OK107>+* (a), *OK107>38377* (b), *OK107>dTDP#5-1* (c), and *OK107>dTDP#18-1* (d). The α, α′, β, β′, and γ lobes were indicated in (a). Note the smaller lobes in (c) and (d). (D) Immunostaining of dTDP in the brains of *OK107>38377* flies with mushroom body-specific knockdown of dTDP expression. Note the significantly reduced signals of dTDP in the mushroom bodies when compared to the *yw* control in Fig. 2A-a. (E) Subcellular distribution of dTDP in the mushroom bodies of the *dTDP*-overexpressing line *OK107>dTDP#5-1*. (a) a set of low magnification pictures; (b) and (c), high magnification pictures of the immunostaining patterns of the *OK107>dTDP#5-1* mushroom body, with (b) from the boxed area in (a) and (c) from the boxed area in (b). Note the presence of cells with mainly cytoplasmic dTDP (the arrow in b) and cells with cytoplasmic dTDP (+) aggregates (the arrowheads). (F) Western blotting analysis of soluble and insoluble proteins in the heads of 3-day old *dTDP*-overexpressing flies. Different fractions of protein extracts were isolated from 3 days-old fly heads as described in Materials and Methods, and analyzed by Western blotting with use of anti-dTDP and anti-tubulin. Note the presence of high molecular weight dTDP species in the urea-soluble fraction of *OK107>dTDP#5-1*. T, total protein; R, RIPA-soluble fraction; U, urea-soluble fraction.

Since human TDP-43 was the major component of the UBIs found in the diseased cells of the central nervous system (CNS) of the ALS and the FTLD-U patients [11], we investigated whether dTDP played a role in the functioning of the *Drosophila* CNS, in particular the mushroom body supporting the olfactory learning of the fruit flies [25], [26]. With use of a home-made anti-dTDP antibody for immunostaining and membrane-targeted GFP (mCD8::GFP) driven by *OK107*-*GAL4* to mark the mushroom bodies, we found that dTDP, similar to the mammalian TDP-43, was broadly distributed in the adult brain with most dTDP molecules located in the cell body, especially in the nucleus (Fig. 2A). We then generated fly lines with mushroom body-specific, dsRNA-mediated knockdown of dTDP expression (*OK107>38377* and *OK107>38379*) and with overexpression of dTDP in the mushroom bodies (*OK107>dTDP#5-1* and *OK107>dTDP#18-1*), respectively, with use of the GAL4-UAS system (Fig. 2).

As seen, Western blot analysis showed that the amounts of dTDP in the adult heads of the two RNAi-knockdown lines were decreased by approximately 46% and 23% (lanes 2 and 3, Fig. 2B), respectively, and those of the two *dTDP*-overexpressing lines were increased by 7 and 3 folds (lanes 4 and 5, Fig. 2B), respectively. All of these four lines survived to the adult stage. Interestingly, however, the structures of the mushroom bodies were changed in the *dTDP*-overexpressing lines although they appeared normal in the *dTDP* knockdown lines (Fig. 2C). In the mushroom bodies, the neurons could be classified into three subtypes according to their projections in the lobes, α/β, α′/β′ and γ. The axons of the α/β neurons bifurcated at the anterior end of the pedunculus to form a vertical α lobe and a horizontal β lobe; and axons of the α′/β′ neurons ran parallel to the α and β lobes to form a vertical α′ lobe and a horizontal β′ lobe. The axons of the γ neurons formed a horizontal γ lobe located anterior to the β and β′ lobes [27]. To visualize the lobe structures, we coexpressed a membrane-target GFP (mCD8::GFP) in the mushroom bodies. While both the control *OK107>+* mushroom bodies (Fig. 2C-a) and the mushroom bodies of the RNAi-knockdown lines, as exemplified for *OK107>38377* (Fig. 2C-b), showed the typical lobe structures, the axon bundles of the mushroom bodies of both *dTDP*-overexpressing lines exhibited shorter lobes (Fig. 2C-c and d). Furthermore, the axonal phenotypes were more heterogeneous and severe in *OK107>dTDP#5-1* (Fig. 2C-c), which had a higher level of dTDP overexpression (Fig. 2B). For instance, 7 out of the 17 *OK107>dTDP#5-1* lines we generated had midline crossing defect (data not shown).

The cellular distribution of dTDP in the mushroom bodies of the above fly lines has also been analyzed by fluorescence immunostaining. As shown in Fig. 2D, the expression of the endogenous dTDP was indeed knocked-down by the transgenic dTDP dsRNAs. On the other hand, the average size of the cell bodies in the mushroom bodies of the *dTDP*-overexpressing flies was somewhat larger, by approximately 1.5-fold in diameter, than that of the control flies (compare Fig. 2E to 2A). Furthermore, while mainly nuclear dTDP was detected in the control mushroom bodies (Fig. 2A), many cells of the mushroom bodies of the *dTDP*-overexpressing lines contained nuclear as well as cytoplasmic dTDP (arrow, Fig. 2E-b). dTDP staining-positive aggregates also existed in the cytoplasm of these cells, as exemplified in Fig. 2E-c (arrowheads, Fig. 2E-c).

In view of the presence of mushroom body cells with dTDP depleted nuclei and cytoplasmic dTDP (+) aggregates similar to those observed in FTLD-U brains, the heads of 3 day-old *dTDP*-overexpressing flies were subjected to Western blotting analysis. As exemplified for *OK107>dTDP#5-1*, significantly higher proportion of dTDP was present in the urea-soluble fraction (lanes 4–6, Fig. 2F) when compared to the control flies (lanes 1–3, Fig. 2F). Furthermore, high-molecular weight dTDP species, presumably the poly-ubiquitinated dTDP, were present in the urea-soluble fraction of the head extract from *OK107>dTDP#5-1* (lane 6, Fig. 2F) but not the control flies (lane 3, Fig. 2F). Thus, the data of Fig. 2E and 2F together indicated that overexpression of dTDP in the *Drosophila* mushroom bodies also led to the formation of urea-soluble, cytoplasmic UBIs, as in the human FTLD-U brains.

### Cognitive behaviours of the *Drosophila* mutant lines with altered dTDP levels in the mushroom bodies

**Figure 3:**
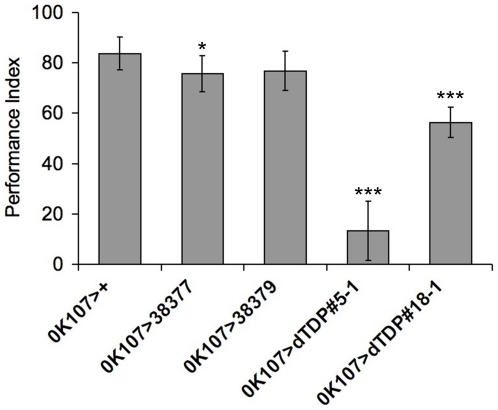
Learning tests of *Drosophila dTDP* knockdown and overexpressing flies. The performance indexes of the control *OK107>+* flies and flies with mushroom body-specific knockdown (*OK107>38377* and *OK107>38379*) or overexpression (*OK107>dTDP#5-1 and OK107>dTDP#18-1*) of dTDP were measured by the olfactory learning tests. Note the dramatic lowering of the indexes of the *dTDP*-overexpressing lines (***, *p*<0.0001) and the relatively small but significant lowering of the index of *OK107>38377* knockdown line (*, *p*<0.05). All value represented mean ± SD. *N* = 8 performance indexes per group.

The learning abilities of the above 4 *dTDP* mutant lines were assayed by the odor avoidance learning test in comparison to the control flies. As shown in Fig. 3, while the lowering of dTDP expression in the mushroom bodies of *OK107>38377* by constitutive RNAi knockdown did not cause obvious defective phenotype of the mushroom bodies (Fig. 2C-b), the performance index of learning of this line showed modest (10%) but statistically significant reductions (*p<0.05*) when compared to the control flies. On the other hand, the olfactory learning abilities of the two *dTDP*-overexpressing lines were severely impaired when compared to the control. Furthermore, parallel to the structural defects of the mushroom bodies (Fig. 2C), the impairment of the learning ability was dTDP dose-dependent: flies with higher dTDP overexpression (*OK107>dTDP#5-1*) showed approximately ∼80% reduction in the performance score when compared to the control flies; on the other hand, the lower level of dTDP overexpression, as in *OK107>dTDP#18-1*, caused only 30% decrease of the learning ability (Fig. 3). The reductions of the learning capabilities of the two independent *UAS-dTDP* lines also suggested that the disruption of the learning ability was not due to a dominant effect of gene disruption at the insertion site of the transgene. The data of Figs. 2 and 3 together indicated that dTDP likely played a role in the learning function of the mushroom bodies. Furthermore, overexpression of dTDP in the mushroom bodies led to gain-of-negative function of dTDP causing abnormal axon lobe phenotype as well as impaired learning ability, a situation similar to FTLD-U (see **Discussion**).

### Larvae and adult phenotypes of *Drosophila* mutants with knockdown or overexpression of dTDP in the motor neurons

**Figure 4:**
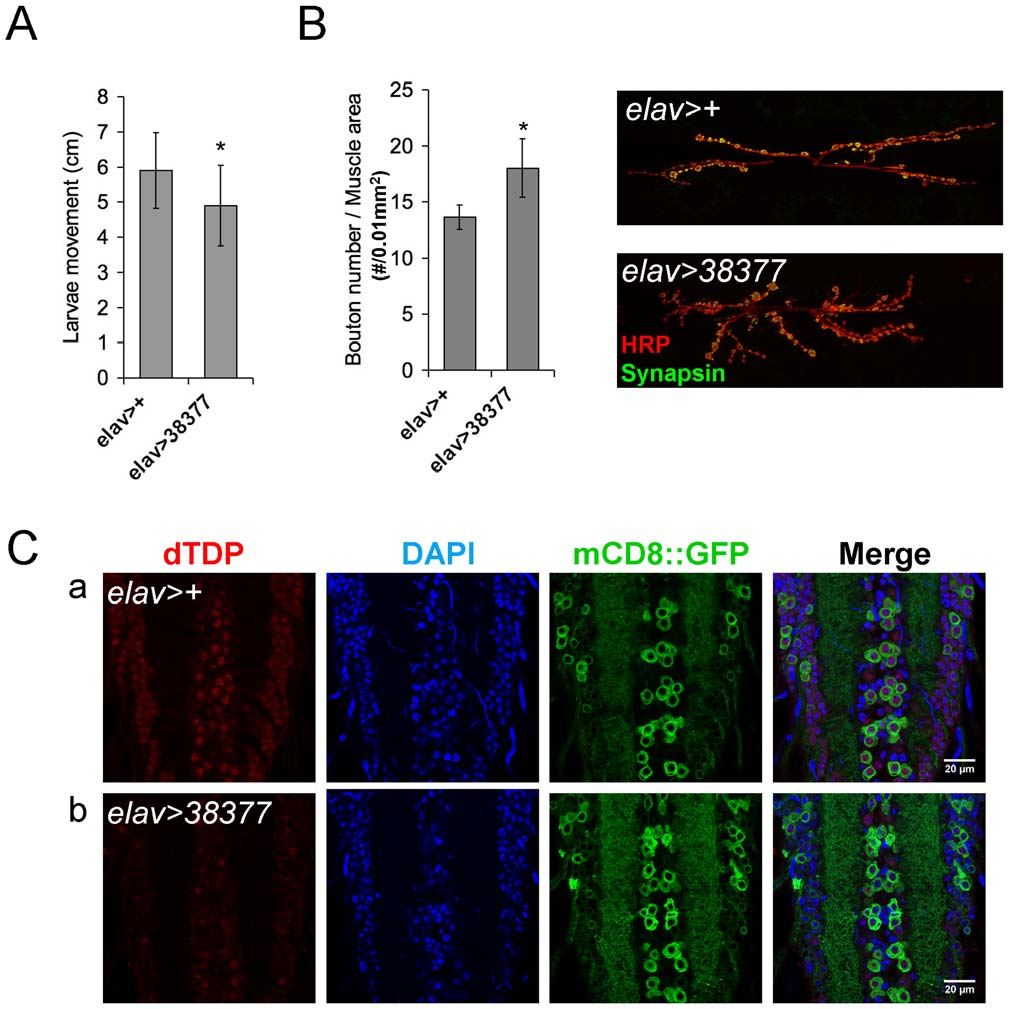
The locomotive activity and NMJ staining of larvae with *dTDP* knockdown in the pan-neurons. (A) Lower locomotive activity of larvae (*elav>38377*) with knockdown of dTDP than the control (*elav>+*) (N = 15, *, *p*<0.05). (B) Quantitative comparison of NMJ bouton numbers of *elav>+* and *elav>38377* larvae after normalization to the total muscle 6/7 areas. Note the higher bouton density of the knockdown larvae (*elav>38377*) than the control (*elav>+*). *, *p*<0.05. The averages of the total bouton numbers are: *elav>+*, 95±14; *elav>38377*, 117±14. The means of the muscle areas are: *elav>+*, 69,937 µm^2^; *elav>38377*, 65,166 µm^2^. (N = 12 in all cases). (C) Whole-mount immunostaining analysis of the larval ventral nerve cords with *dTDP*-knockdown in the pan-neurons. (a), control flies *elav>+*; (b) *elav>38377*. Red, dTDP; blue, DAPI; green, mCD8::GFP. Note that the pan-neuron-driven dsRNA did not completely knock down the dTDP expression since there were still some dTDP signals in the larval ventral nerve cords of *elav>38377*.

In view of the loss-of-function phenotype of the locomotive ability of flies with whole-body knockout of dTDP expression (Fig. 1; [23]) and the disease phenotypes caused by overexpression of human hTDP-43 in *Drosophila* [19], we have tested whether the pathway(s) leading to ALS disease pathology was conserved in the fruit flies by knockdown or overexpression of dTDP in the motor neurons of *Drosophila*. We first used the motor neuron-specific driver (*D42*-*GAL4*) to knockdown the endogenous dTDP in the motor neurons. However, the resulting flies did not exhibit locomotion defect, possibly due to inefficient knockdown of the dTDP. We then used the pan-neuron driver *elav*-*GAL4* to knockdown dTDP. As shown in Fig. 4A, the moving abilities of larvae from *elav>38377* were lower than the control larvae (*elav>+*). Furthermore, similar to the *dTDP*-null fly line *dTDP^ex26^* described in Fig. 1, the NMJ boutons of *elav>38377* larvae were also higher than the control larvae (Fig. 4B). Although these differences between the control (*elav>+*) and the *dTDP*-knockdown larvae (*elav>38377*) were significant (*p*<0.05), it was less than those between the control (*yw*) and the *dTDP*-null mutant *dTDP^ex26^* as shown in Fig. 1C and 1D. This might result from the inefficient knock-down of dTDP in the pan-neurons. Indeed, unlike in *dTDP^ex26^* (Fig. 1B), some dTDP signals were present in the ventral nerve cords of the *elav>38377* flies (Fig. 4C). Finally, we also knocked down dTDP expression in the muscle areas as described in the Materials and Methods, but this step did not cause any change in either the bouton numbers or the larval movement (data not shown).

**Figure 5:**
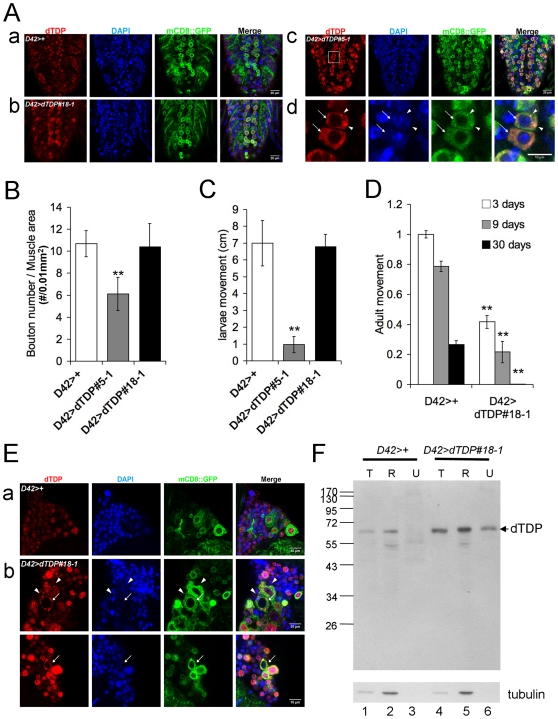
Pathologies of flies with motor neuron-specific overexpression of dTDP. (A) Whole-mount immunostaining analysis of the larval ventral nerve cords. (a), control flies *D42>+*; (b) *D42>dTDP#18-1*; (c) *D42>dTDP#5-1*; (d) magnified pictures of the boxed area in (c). Red, dTDP; blue, DAPI; green, mCD8::GFP. Note the predominant nuclear localization of dTDP in (a). (b), in the low-level *dTDP*-overexpressing flies (*D42>dTDP#18-1*), dTDP was distributed in both the nucleus and cytosol. (c) and (d), in the high-level *dTDP*-overexpressing flies (*D42>dTDP#5-1*), dTDP became predominantly localized in the cytosol (arrows in d) and often formed dTDP (+) aggregates (arrowheads in d). (B) Histograph of the densities of the bouton numbers. NMJ staining of the third instar larvae was carried out for *D42>+*, *D42>dTDP#5-1*, and *D42>dTDP#18-1*, and the densities of the bouton numbers were counted and compared. Note that a decrease of the bouton number was observed only in the high-level *dTDP*-overexpressing fly line (*D42>dTDP#5-1*), **, *p*<0.001. The averages of the total bouton numbers are: *D42>+*, 80±4 (N = 20); *D42>dTDP#5-1*, 46±15 (N = 12); *D42>dTDP#18-1*, 74±17 (N = 20). The means of the muscle areas are: *D42>+*, 75,925 µm^2^; *D42>dTDP#5-1*, 56,177 µm^2^; *D42>dTDP#18-1*, 71,189 µm^2^. (C) Larvae movement assay. Parallel to data of (B), only the larvae with high dTDP overexpression (*D42>dTDP#5-1*) showed severe movement defect. (D) and (E) Pathogenesis of adult flies with motor neuron-specific overexpression of dTDP. (D) Movement defect of fly line *D42>dTDP#18-1*. Phototaxis was performed at three different ages of the adult flies and the movement indexes were normalized to that of the control line *D42>+*. Note the age-dependent declines of the performance of the *dTDP*-overexpressing flies. *N* = 5, **, *p*<0.001. (E) Whole-mount immunostaining of the thoracic ganglia of day-9 adult flies. Red, dTDP; blue, DAPI; green, mCD8::GFP. (a) Control flies (*D42>+*). Note the predominant nuclear localization of dTDP. (b) *D42>dTDP#18-1* flies. Two regions under the confocal microscope are shown. Note the presence of cells with mainly cytoplasmic distribution of dTDP, as exemplified by cells pointed with the arrows. The dTDP (+) aggregates in the cytoplasm are exemplified by the arrowheads. (F) Western blotting analysis of soluble and insoluble proteins in the heads and thoraces of 13-day old *D42>dTDP#18-1* flies with the motor neuron-specific overexpression of dTDP. Different fractions of the protein extracts were isolated from the heads and thoraces as described in the Materials and Methods, and analyzed by Western blotting with use of anti-dTDP and anti-tubulin. T, total protein; R, RIPA-soluble fraction; U, urea-soluble fraction. Note the presence of dTDP in the urea-soluble fraction from *D42>dTDP#18-1* but not *D42>+*.

Two independent lines (*D42>dTDP#5-1* and *D42>dTDP#18-1*) with dTDP overexpression in the motor neurons were also generated with use of the GAL4-UAS system. Immunostaining was then used to examine the levels and distribution patterns of dTDP in the larval ventral nerve cords of these two mutant lines in comparison to the *D42>+* control flies. As seen in Fig. 5A-a, the endogenous dTDP of the wild type was of relatively low level and mostly in the nucleus. On the other hand, both overexpressing lines had elevated levels of dTDP than the control (compared b and c to a of Fig. 5A). Furthermore, in the two *dTDP*-overexpressing lines, the dTDP molecules appeared to be translocated from nucleus to the cytoplasm (Fig. 5A-b and c) in a dTDP dose-dependent manner. In *D42>dTDP#18-1*, the dTDP signals were present in both the nucleus and cytoplasm (Fig. 5A-b); in line *D42>dTDP#5-1* which had a higher level of dTDP overexpression in the motor neurons (Fig. 5A-c), the dTDP signals were mostly detected in the cytoplasm (arrows, Fig. 5A-d) and some cells even contained dTDP (+) aggregates (arrowheads, Fig. 5A-d). Interestingly, the survivals of these flies were also dTDP dose-dependent, with all of the flies of *D42>dTDP#5-1* dying at early pupal stage while the average life span of the *D42>dTDP#18-1* flies being 18 days.

We next carried out NMJ immunostaining and locomotion tests of larvae of *D42>dTDP#5-1* and *D42>dTDP#18-1*. In interesting contrast to the increase of the bouton number of NMJ upon knockout of the dTDP expression (Fig. 1 and 4), the motor neuron-specific overexpression of dTDP caused a dose-dependent decrease of the bouton number of NMJ (Fig. 5B). A dose-dependent decrease of the number of axon branches in the mutant flies was also observed (data not shown). Significantly, the dose-dependence of the structural changes of the NMJ was parallel to that observed in the larval locomotion test, in which *D42>dTDP#18-1* behaved similarly as the control larvae, but movement of the larvae of the higher *dTDP*-overexpressing line *D42>dTDP#5-1* was severely impaired (Fig. 5C). We have also examined the effects of dTDP overexpression on the locomotor activity of the adult flies. For this, *D42>dTDP#18-1* flies were analyzed by the phototaxis assay since they could survive till the adult stage as mentioned above. Remarkably, while the locomotive behavior of these flies was not affected at the larval stage (Fig. 5C), the adult *D42>dTDP#18-1* flies exhibited lower mobility than the control flies in an age-dependent manner (Fig. 5D). At day 3, the *dTDP*-overexpressing flies had only 40% movement ability as the control flies. At the age of 9 days, the movement ability further decreased down to 27% of the control flies. Strikingly, at day 30, the *D42>dTDP#18-1* flies almost completely lost their locomotor activities. The control flies, on the other hand, exhibited only a 20% decline of their movement abilities as they aged from 3-day to 9-day and at the age of 30 days, they still kept 20% of the movement abilities (Fig. 5D).

To see if the locomotion defect of the adult *D42>dTDP#18-1* flies was also associated with abnormal cellular distribution patterns of dTDP, as that observed for the *dTDP*-overexpressing larvae (Fig. 5A), we dissected the adult thoracic ganglia and performed immunostaining assay. Similar to the larval ventral nerve cords (Fig. 5A-a), the endogenous dTDP was mostly in the nucleus (Fig. 5E-a). In the *dTDP*-overexpressing flies, however, the dTDP molecules were distributed in both the nucleus and cytoplasm (Fig. 5E-b). Also, dTDP could be found only in the cytosol in some of the cells (arrows in Fig. 5E-b). Furthermore, cytoplasmic dTDP (+) aggregates could also be detected in these cells (arrowheads, Fig. 5E-b). Significantly, the observation of cytosolic dTDP (+) aggregates in the immunostaining experiments of Fig. 5E was paralleled by the presence of dTDP in the urea-soluble fraction of extracts from the heads/thoraces of *D42>dTDP#18-1* flies, as exemplified in the Western blot of Fig. 5F. The results from experiments exemplified in Figs. 4 and 5 suggested that dTDP also played an essential role in the motor function of *Drosophila* at both the larval and adult stages, as modulated in part through the regulation of biogenesis of the boutons at NMJ. Furthermore, mis-regulation of the homeostatic concentration of dTDP, eg, elevation of the amount of dTDP, could impair this function and lead to the development of ALS-like phenotype in the fruit flies.

## Discussion

In this study, we have investigated the functions of the *Drosophila* dTDP protein, the ortholog of the mammalian TDP-43. In view of the close association of TDP-43 in the pathogenesis of human FTLD-U and ALS, we have also tested the consequences of overexpression of dTDP in *Drosophila* in order to establish new fly models of human neurodegenerative diseases with TDP-43 (+) UBIs. It should be mentioned that overexpression of human hTDP-43 in *Drosophila* has been carried out previously by others [19], [24]. The current study allows the test of whether the mutant phenotypes of the fruit flies observed in those studies are due to the toxicity of the human hTDP-43 or as the result of elevation of the level of the total amount of TDP-43. Our data show that dTDP not only functions in the early development and locomotor activities of the fruit flies, as observed previously [23], but it is also important for synaptogenesis and learning of *Drosophila*. They also indicate that *Drosophila* with overexpression of dTDP could be developed into working disease models if the dose of the exogeneous dTDP is appropriately controlled. Finally, the analysis of *Drosophila* lines with knockdown and overexpression of dTDP suggests that phenotypes of FTLD-U and ALS with TDP-43 (+) UBIs are caused by a combination of loss-of-TDP-43 function and gain-of-negative function of TDP-43.

The developmental function of dTDP has been examined by generation of *dTDP*-null mutant *dTDP^ex26^* using P-element excision, which exhibits early lethal phenotype with locomotion defects in the larvae as well as the adult flies (Fig. 1). This phenotype is similar to those reported for a different P-element excision *Drosophila* mutant [23]. Interestingly, in *TDP-43* knock-out mouse models, loss of mouse mTDP-43 expression results in lethality of the mice between embryonic days 3.5 and 6.5 [28], [29], which appears to be due to inability of the inner cell mass to proliferate [29]. These studies together point to a conserved role of TDP-43 in the early development of the animals, the detailed molecular and cellular basis of which wait to be investigated.

In view of the impairment of learning and memory in FTLD-U patients with TDP-43 (+) UBIs [30], the apparent association of mTDP-43 with molecules/structures implicated in the regulation of neuronal plasticity [5], and the abundant expression of dTDP in the *Drosophila* brains (Fig. 2A; data not shown), we have also studied the role of dTDP in the *Drosophila* CNS by both knockdown and overexpression of dTDP in the mushroom bodies (Fig. 2). Remarkably, flies of one line with constitutive mushroom body-specific knockdown of dTDP (*OK107>38377*) have normal life span and apparently normal mushroom bodies even at the age of 30-day old (Fig. 2C-b and data not shown), but they exhibit decreased learning capability in the olfactory odor avoidance learning test (Fig. 3). On the other hand, constitutive overexpression of dTDP in the mushroom bodies leads to the development of an abnormal axonal structure with smaller axonal lobes and midline crossing defect (Fig. 2C-c and 2C-d, and data not shown). As expected from the known function of the mushroom bodies in learning and memory of the flies [26], [31], dose-dependent impairment of the learning capabilities of these *dTDP*-overexpressing lines is apparent (Fig. 3). These data together support the scenario that the TDP-43 protein functions in the learning process of the fruit flies and, by implication, the mammals. Furthermore, impairment of the learning in some of the FTLD-U cases with TDP-43 (+) UBIs is likely due to a dominant negative effect by the elevated amount of the TDP-43 protein, as also suggested by the study of a FTLD-U transgenic mouse model with transgenic overexpression of TDP-43 under the control of a CamKII promoter [20].

dTDP also appears to be required for functioning of the motor neurons, as reflected by the locomotion defects of the larvae of *dTDP^ex26^* null mutant (Fig. 1C) and *elav>38377* (Fig. 4A). Significantly, the locomotion defects in both cases are accompanied with approximately 30% increase of the bouton numbers at NMJ, when compared to the wild type (Fig. 1D and 4B). It should be noted that while the phenotype of locomotion defect of *dTDP^ex26^* larvae is similar to two other P-element excision lines of *Drosophila* [23], these latter lines display lower bouton number at NMJ [23]. This difference between the two studies might result from the different genetic backgrounds and/or different deletions of the *dTDP* gene of these lines. In interesting contrast to our *dTDP^ex26^* and *elav>38377* lines, larvae with overexpression of dTDP (Fig. 5) or hTDP-43 [19] in the motor neurons, while also being defective in locomotion, have decreased bouton numbers at NMJ instead. Several points are worthy to note here. First, the locomotion defects as caused by overexpression of the wild type dTDP (Fig. 5) or hTDP-43 [18], [19] in the motor neurons are similar to those of the transgenic mice with overexpression of wild type mTDP-43 or hTDP-43 under the control of different promoters [20], [21]. Second, similar to ALS, development of the locomotion defects in flies with overexpression of dTDP is age-dependent, as exemplified by the *D42>dTDP#18-1* line (Fig. 5D). Third, the opposite effects of depletion of dTDP expression and overexpression of dTDP in the motor neurons of *Drosophila* suggest that dTDP plays a role in the biogenesis of the boutons of the fly NMJ and, by implication, the synapse formation or differentiation of the vertebrate nervous system. By further implication, the phenotypes of ALS and FTLD-U, are likely due, at least in part, to the failure of normal synaptogenesis on the spinal motor neurons and cortical neurons, respectively.

Notably, the abnormal axon lobe phenotypes of flies with overexpression of dTDP in their mushroom bodies are accompanied with the subcellular redistribution of dTDP molecules leading to the appearance of cells with cytoplasmic dTDP (+) aggregates and dTDP-depleted nuclei (Fig. 2E-c), and aberrantly processed dTDP molecules (Fig. 2F), all of which are reminiscent of diseased cells of FTLD-U with TDP-43 (+) UBIs. In interesting parallel, overexpression of dTDP in the motor neurons leads to reduced life span and locomotion deficiency of the larvae (Fig. 5C) and adult flies (Fig. 5D) with the appearance of cells containing dTDP-depleted nuclei and/or cytosolic dTDP aggregates in the larval ventral nerve cords (Fig. 5A-d) as well as in the adult thoracic ganglia (Fig. 5E-b). Furthermore, dTDP dose-dependent severity of the pathogenesis, e.g., shortening of the life span and locomotive dysfunction, has been observed (Fig. 5C). These fruit fly data are in interesting parallel to reports of FTLD-U and ALS patients with elevated levels of TDP-43 [32], [33] and several transgenic rodent models with overexpression of the mammalian TDP-43 proteins [20], [21], [34], [35]. In particular, transgenic hTDP-43 mice have reduced life span and they develop paralysis and spasticity, the severities of which depend on the levels of the overexpressed hTDP-43 [21]. On the other hand, overexpression of mTDP-43 in the mouse forehead leads to the generation of a FTLD-U mouse model [20]. The transgenic fly and mouse studies together provide support for a causative role of the elevated levels of the endogenous hTDP-43 in patients with FTLD-U and ALS.

In summary, this study has demonstrated the requirement of dTDP for development and for neuronal functioning, in learning as well as in locomotion, of the fruit flies. The feasibility of the generation of fly models of neurodegeneration diseases with TDP-43 (+) UBIs by overexpression of the homologous *Drosophila* dTDP in appropriate tissues/cells has also been shown. Thus, in addition to the protein sequences and general structure of the hTDP-43/dTDP [2], [9], [36], the biological pathways responsible for the function and dysfunction of mammalian TDP-43 are also well conserved in *Drosophila*. Fly models with overexpression of the homologous dTDP likely will have certain advantages when compared to the ones with overexpression of the heterologous hTDP-43, for the basic and translational research of neurodegenerative diseases with TDP-43 proteinopathies.

## Materials and Methods

### Manipulation of dTDP levels in *Drosophila*

Fly stocks were maintained in standard medium at 25°C and 60% humidity under a 12-h light-dark cycle. The pan-neuron GAL4 (*elav-GAL4*) flies, motor neuron-specific GAL4 (*D42-GAL4*) flies, mushroom body-specific GAL4 (*OK107-GAL4*) flies and P-element insertion mutation stock of *dTDP*: *y^1^ w^67c23^; P{SUPor-P}TBPH^KG08578^* (*KG08578*) were obtained from the Bloomington *Drosophila* Stock Center. The muscle-specific GAL4 flies, *MHC-GAL4*, were kindly provided by Dr. Chien's lab at Institute of Molecular Biology (IMB), Academia Sinica. The transgenic RNAi stocks *VDRC38377* and *VDRC38379* were obtained from the Vienna *Drosophila* RNAi Center. The P-element excision of *KG08578* was carried out by the standard P-element mobilization approach. Homologous mutants at the early development stage were created with use of a green balancer.

*pUAST-dTDP* was constructed by subcloning of *dTDP* cDNA from EST clone *GH09868* into the *pUAST* vector. To knockdown dTDP in neuron cells, the virgin females of *elav-GAL4* were crossed with males of the RNAi line, *VDRC38377*. The virgin females of the RNAi stocks or *pUAST-dTDP* were crossed with males of *OK107-GAL4* to generate flies with mushroom body-specific knockdown of dTDP or dTDP overexpression. For knockdown or overexpression of dTDP in the motor neurons, the UAS lines were crossed with *D42-GAL4*. Flies with muscle-specific knockdown of dTDP were obtained by cross between the virgin females of *VDRC38377* and the males of *MHC-GAL4*. To perform the rescue experiments, the virgin females of *dTDP^ex26^,UAS-dTDP#18-1/T(2,3)CyO:TM6B,Tb^1^* were crossed with the males of *dTDP^ex26^,D42-GAL4/T(2,3)CyO:TM6B,Tb^1^*. The excision line *dTDP^ex26^* was crossed with *yw* for five generations and all the other stocks described above, were equilibrated by six generations of out-cross to the *w* background.

### RNA isolation and RT-PCR

Total RNAs were isolated from *Drosophila melanogaster* at different stages by using the Trizol reagent (Invitrogen). For analysis of the expression levels of dTDP, 1 µg of the total RNAs were reverse transcribed with SuperScript™ II Reverse Transcriptase (Invitrogen) using oligo (dT) primers. The primers used for PCR reactions are: dTDP-F, 5′-CCATGGATTTCGTTCAAGTGTCG-3′; and dTDP-R, 5′-TTAAAGAAAGTTTGA CTTCTCCGC-3′.

### dTDP Antibody Generation

A cDNA fragment encoding the C-terminus of dTDP (amino acid 307–531) was amplified by PCR reaction and cloned into BamHI/XhoI sites of pRSET vector. Expression of the protein and immunization of the rabbits were done by the commercial supplier (LTK BioLaboratories, Taiwan).

### Immunostaining assay

For muscle staining and analysis of the NMJ phenotypes, wandering late third instar larvae were dissected and the tissues were incubated in a fixative solution (4% paraformaldehyde in calcium-free saline) for 20 minutes. The tissues were then blocked with 10% normal goat serum at room temperature for 1h. The primary antibodies used were against dTDP (1∶200), synapsin (3C11, 1∶100; DHSB) and HRP (goat anti-HRP conjugated with TRITC, 1∶100; Jackson ImmunoResearch), respectively. For secondary antibody, goat anti-rabbit conjugated to Alex Fluor 647 or goat anti-mouse conjugated to Alex Fluor 647 was used (Molecular Probes). FITC-conjugated Phalloidin was used for muscle staining.

For staining of the larval ventral nerve cords, adult brains, and adult thoracic ganglia, larvae or adult females were dissected in PBS, fixed with 4% paraformaldehyde in PBS with 0.25% Triton-X-100, and then irradiated using a microwave oven (Pelco BioWave, Pelco International). After blocking with 10% normal goat serum at room temperature for 1 h, the tissues were incubated overnight at room temperature with rabbit polyclonal anti-dTDP (1∶200). After washing, the tissues were incubated overnight at room temperature with biotinylated anti-rabbit antibody (1∶ 250; Molecular Probes) and rat anti-elav antibody (1∶ 200; DHSB). They were then washed again and incubated overnight with streptavidin-Alexa-647 antibody (1∶ 1000; Molecular Probes) and Alexa-546 conjugated goat anti-rat antibody (1∶ 500; Molecular Probes).

The confocal images were acquired using a Zeiss LSM 510 Meta or LSM710. The images for quantification of the NMJ bouton numbers were from a projection of the z-sections. To quantify the NMJ muscle area, the images were analyzed using Zeiss LSM Image Examiner.

### Locomotor activity assay

The locomotor activity of the adult flies was measured in a dark room using a countercurrent apparatus as described [37]. During the test, groups of 50 flies (25 males and 25 females) of the ages 3 days, 9 days and 30 days, respectively, were given 15 sec to move toward a white light source. They were then tapped down and moved to the next tube of the apparatus. After five runs, the distributions of the flies was measured, with the most active flies running into the distal tubes 5 times receiving a score of five and the least active flies staying in the original start tube throughout the experiment receiving a score of zero. All the scores were then normalized against the average score of the 3 day-old control flies.

To measure the larval locomotor activity, 20 larvae per genotype were transferred to a Petri dish coated with 1% agarose, and their behaviors were monitored for 2 minutes. The average distance of the larval trails of each group was then determined with use of an ImageJ software.

### Odor avoidance learning test

The abilities of the olfactory associative learning of the flies were measured by training 2∼5-day old adult flies in a T-maze following the classical conditioning procedure [38]. Approximately 100 flies were trapped in an electrifiable, copper grid-covered training chamber, and then exposed to two odors, 3-octanol (OCT) and 4-methyl-cyclohexanol (MCH), sequentially. The flies were received electrical shocks during exposure to the first odor (conditioned stimulus, CS+; MCH or OCT) but not the second (CS−; OCT or MCH). After one cycle of training, the flies were gently tapped into an elevator-like compartment to assay for their learning of the odor avoidance. The flies were then transported to the choice point of a T-maze, in which they were exposed to two converging currents of air (one carrying OCT, the other MCH) from opposite arms of the T-maze. After 2 minutes, the flies were transferred from each collection tube to a polypropylene tube, and then counted under anesthesia condition. A performance index was then calculated as the number of flies avoiding CS+ minus that avoiding CS−, divided by the total number of flies.

### Solubility Analysis

To examine the solubility profiles of dTDP, sequential extractions were performed as described by Winton et al. [39]. For comparison of *OK107>+* and *OK107>dTDP#5-1*, 100 *Drosophila* heads from 50 males and 50 females of the age of 3 days were collected. For comparison of *D42>+* and *D42>dTDP#18-1*, the heads and thoraces from 20 males and 20 females of the age of 13 days were collected. All samples were lysed in cold RIPA buffer (0.1% SDS, 1% Nonidet P-40, 0.5% sodium deoxycholate, 150 mM NaCl, 50 mM Tris-HCl, pH 7.9) containing protease and phosphatase inhibitors (1 mM phenylmethylsulfonyl fluoride, 1 mM NaF, 1 mM sodium orthovanadate, and a mixture of protease inhibitors, Roche), and then sonicated. The protein lysates were first cleared by centrifugation to generate the RIPA-soluble samples. The pellets were then washed four times with RIPA buffer and extracted with the urea buffer (7 M urea, 2 M thiourea, 4% CHAPS, 30 mM Tris, pH 8.5), sonicated, and centrifuged. For preparation of the total extracts, the *Drosophila* samples were lysed directly in the urea buffer.
